# Optic nerve and susceptibility imaging at asymptomatic stage of multiple sclerosis: impact and predictive value

**DOI:** 10.1093/braincomms/fcag020

**Published:** 2026-01-21

**Authors:** Jean-Christophe Lafontaine, Cécile Bordier, Julien Labreuche, Tifanie Alberto, Bruno Lemarchant, Hélène Zéphir, Olivier Outteryck

**Affiliations:** Centre de Ressources et Compétences Sclérose En Plaques (CRCSEP) de Lille, Department of Neurology, CHU Lille, Lille F-59000, France; Univ. Lille, INSERM, CHU Lille, U1172, Lille F-59000, France; Univ. Lille, CNRS, INSERM, CHU Lille, Institut Pasteur de Lille, US 41 - UAR 2014, Lille F-59000, France; Department of Biostatistics, CHU Lille, Lille F-59000, France; Centre de Ressources et Compétences Sclérose En Plaques (CRCSEP) de Lille, Department of Neurology, CHU Lille, Lille F-59000, France; Centre de Ressources et Compétences Sclérose En Plaques (CRCSEP) de Lille, Department of Neurology, CHU Lille, Lille F-59000, France; Univ. Lille, INSERM, CHU Lille, U1172, Lille F-59000, France; Centre de Ressources et Compétences Sclérose En Plaques (CRCSEP) de Lille, Department of Neurology, CHU Lille, Lille F-59000, France; Univ. Lille, INSERM, CHU Lille, U1172, Lille F-59000, France; Univ. Lille, INSERM, CHU Lille, U1172, Lille F-59000, France; Department of Neuroradiology, CHU Lille, Lille F-59000France

**Keywords:** radiologically isolated syndrome, multiple sclerosis, optical coherence tomography, optic nerve MRI, paramagnetic rim lesion

## Abstract

Optic nerve is now included as a fifth typical location in multiple sclerosis diagnosis criteria. The radiologically isolated syndrome represents the earliest stage of multiple sclerosis. Previous optical coherence tomography studies in this asymptomatic context reported no or slight retinal thickness difference compared to healthy subjects. Frequency of asymptomatic optic nerve lesions has never been evaluated at this stage of the disease. Susceptibility-weighted imaging findings on brain MRI are incorporated in the recent revised multiple sclerosis diagnostic criteria (2024) through the ‘central vein sign’ and ‘paramagnetic rim lesion’ parameters but for the diagnosis of asymptomatic form, ‘paramagnetic rim lesion’ are not included. In this study, we aim to measure the frequency of optic nerve lesions in radiologically isolated syndrome and to evaluate their impact on retinal thicknesses. Second, we aim to evaluate the association of optic nerve lesion and susceptibility-weighted imaging parameters with the disease course.

This retrospective cohort study collected data (August 2020 to December 2024) on patients with radiologically isolated syndrome at Lille (France). MRI was performed at baseline and every year. Optic nerves were studied using MRI and optical coherence tomography performed on the same day by measuring retinal thickness intereye difference. Clinical examination was performed every 6 months.

We included 32 untreated patients (63 eyes; one eye excluded due to the fortuitous discovery of an ocular melanoma). Nine optic nerves showed lesions on MRI in the orbital or canalicular part. These eyes had a thinner peripapillary retinal nerve fibre layer compared to eyes without optic nerve lesions on MRI (median = 87.4 µm versus 96.8 µm, *P* = 0.003). No association was found between peripapillary retinal nerve fibre layer thickness and quantitative MRI parameters as optic radiations T2 lesions or primary visual cortex volumes.

During follow-up (median: 22.1 months), three patients converted to relapsing multiple sclerosis and two patients to progressive multiple sclerosis. Among them, 60% had an optic nerve lesion (versus 25.9%) and 60% had at least one paramagnetic rim lesion (versus 25.9%). In total, 24 patients fulfilled dissemination in time and space according to the revised multiple sclerosis diagnostic criteria (2024).

As in clinically isolated syndrome and clinically definite MS, silent optic nerve lesions seem to be the main cause of subclinical retinal neuro-axonal loss at radiologically isolated syndrome stage. Our results suggest that patients with paramagnetic rim lesion or optic nerve lesion might present a higher risk of clinical conversion.

## Teaser

Lafontaine *et al*. report that optic nerve (21.9%) and paramagnetic rim (31.5%) lesions are frequently observed in patients with asymptomatic multiple sclerosis, particularly in those who will experience clinical conversion. Optic nerve lesions were associated with a significant retinal thinning, suggesting that this is the main cause of retinal axonal loss.

## Graphical Abstract

**Graphical Abstract:**
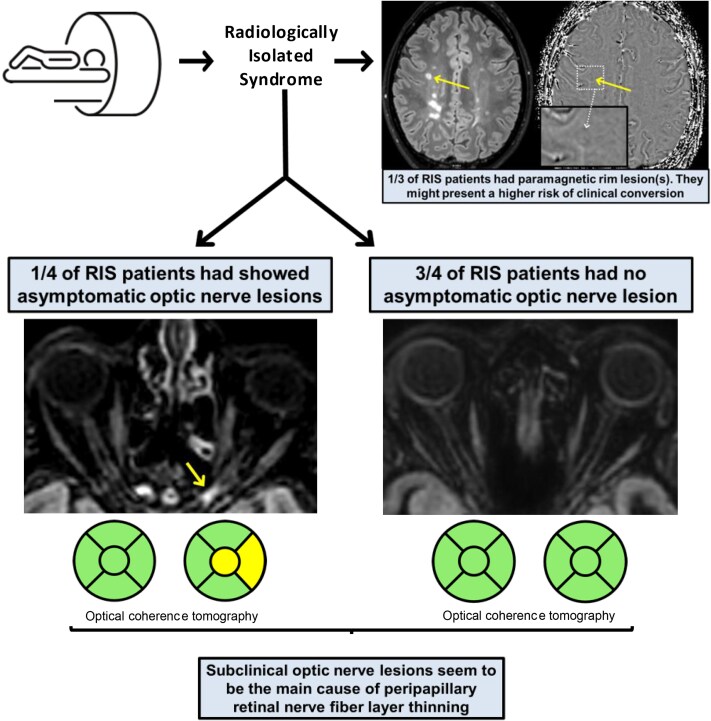

## Introduction

Patients undergoing MRI for various reasons can present with incidental lesions highly suggestive of multiple sclerosis (MS) without clinical abnormalities. The radiologically isolated syndrome (RIS) as it is defined, is a diagnosis established according to validated criteria recently modified.^1^ RIS is considered to be the preclinical stage of MS and the earliest stage of MS considering new MS criteria to be published. The visual pathways are a relevant model for measuring neuroaxonal degeneration and its relationship with acute and chronic demyelination lesion in multiple sclerosis, regardless of the stage of the disease. In addition, visual pathways damage, specifically through symptomatic and asymptomatic lesions of the optic nerve, have recently been added to the diagnostic criteria for MS.^2^

Previous optical coherence tomography (OCT) studies reported no retinal thickness difference between healthy control (HC) subjects and people with RIS suggesting no evidence or early retinal degeneration at the RIS stage.^3,4^ However, some others reported slightly thinner macular ganglion cells inner plexiform layer (GCIPL),^5,6^ temporal peripapillary retinal nerve fibre layer (pRNFL)^6^ or global pRNFL thicknesses.^5^ Retinal OCT parameters like pRNFL, GCIPL and inner nuclear layer (INL) thicknesses/volumes have also been proposed as prognosis factors in RIS.^5-7^ Spinal cord and infratentorial lesions, which are known to be associated with worse prognosis in MS^8,9^ have been independently associated with lower GCIPL thickness in RIS.^4^ These retinal thickness alterations support the presence of a more diffuse disease and may be associated with brain damage through a retrograde trans-synaptic degeneration^10^ at the earliest stage of MS. In RIS, all lesions are considered asymptomatic and literature on OCT clearly demonstrated that neurodegeneration has already begun. Our group previously reported multimodal studies showing the major role of asymptomatic optic nerve lesion(s) on asymptomatic retinal neuroaxonal degeneration at the earliest clinical stage of MS, the clinically isolated syndrome (CIS)^11^ and at a later stage of clinically definite MS.^12^ Asymptomatic optic nerve lesion on MRI are frequent ranging from one third in patients with CIS^13^ to three quarters of patients with MS.^12,14^ Radiologically isolated syndrome represents the earliest stage of MS and up to now there is no study evaluating prevalence of asymptomatic optic nerve lesion on MRI and its role on retinal neurodegeneration.

In RIS, several risk factors of clinical conversion have been described such as spinal cord lesion, age < 37 years, infra-tentorial lesions or oligoclonal bands (OCBs).^15-17^ Revised MS diagnostic criteria have been recently presented.^2^ Optic nerve is now part of the typical locations of MS demyelinating lesions. Additional MRI features have been introduced for the diagnosis of MS, such as the ‘central vein sign’ (CVS)^18^ and the ‘paramagnetic rim lesion’ (PRL).^19^ However, paramagnetic rim lesion is not part of the diagnosis of asymptomatic MS and predictive value of optic nerve lesion needs to be evaluated at RIS stage.

Objectives of our study are to assess the impact of asymptomatic optic nerve lesions on MRI on retinal layers thickness at the preclinical stage of MS (RIS) and to describe the association of new biomarkers included in the most recent MS diagnosis criteria^2^ with the disease course.

## Material and methods

### Design and inclusion criteria

This is a monocentric retrospective cohort study conducted on data collected between August 2020 and December 2024, in Lille University Hospital (France). All patients were followed at the Lille MS centre (FR). Non-opposition to use clinical and radiological data was obtained from each included patient (MR004-MISSYVV; DEC22-140; https://www.chu-lille.fr/rgpd-recherche). Included patients presented a RIS according to the revised diagnostic criteria by Lebrun-Frenay *et al*. in 2023.^20^ Patients were included at baseline, i.e. at the time of the first concomitant retinal OCT and optic nerve/brain/spinal cord MRI. No patient was treated at the time of OCT/MRI evaluation.

Data on gender, age at OCT/MRI work-up, age at RIS onset, reason of the first MRI scan were collected. During follow-up and as part of care, brain and spinal MRI was performed every year, and a clinical examination was carried out every 6 months. Data on the first relapse, disease progression and new brain/spinal cord T2 lesion(s), were collected up to the last follow-up. Patients who develop a relapse or a disease progression during the follow-up were classified in the ‘clinically converting group’. Others remain classified as RIS. Patients who develop new brain/spinal cord T2 lesion(s) during the follow-up were classified in the ‘MRI converting group’.

### MRI

Brain and spinal cord MRI was performed on a 3T MRI (Achieva; Philips) as previously published,^11^ including an additional susceptibility-weighted imaging (SWI) sequence 3D-T2*EPI (3D-FFE [Fast Field Echo] EPI [echo planar imaging] 3D multishot technique, EPI [factor 15], TR/TE = 66/35 ms, axial acquisition, voxel size 0,65 × 0,65 × 0,65 mm, FOV 250 × 250 × 166, number of slices 256, fat suppression). Thus, the brain imaging protocol (32-channel array head coils) included the following sequences: 3D-T1-FFE (gradient echo), 3D-DIR, 3D-T2*EPI (SWI), 3D-FLAIR, 3D-T1-TSE with gadolinium. During optic nerve imaging, patients were asked to close their eyes and avoid eye movements as much as possible. The spinal cord imaging protocol included: sagittal T1-TSE with gadolinium and T2-SPAIR (spectral attenuated inversion recovery) of the whole spinal cord.

For each patient, we recorded the presence/absence of T2-weighted lesions in each of the five anatomical areas for demyelinating multiple sclerosis lesions (optic nerve, cortical/juxtacortical, periventricular, infratentorial and spinal cord), the presence/absence of gadolinium-enhancing lesions.

**Figure 1:**
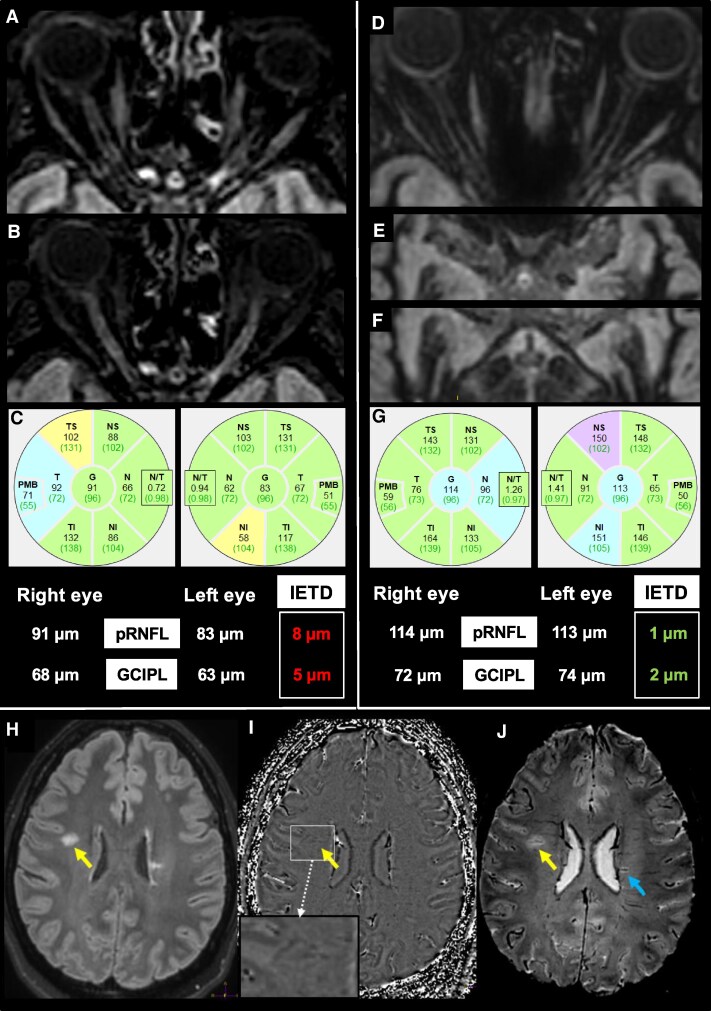
**Brain/optic nerves MRI and OCT finding in patients with radiologically isolated syndrome.** (**A, B**) A patient with RIS presented with a left canalicular optic nerve lesion on 3D-DIR (**A**) and 3D FLAIR (**B**) axial reconstructions. OCT also showed a decreased pRNFL and GCIPL on the left eye with pRNFL IETD of 8 µm and GCIPL-IETD of 5 µm, suggesting the presence of a left optic nerve lesion (**C**). (**D–G**) A patient with RIS presented without any lesion on the anterior visual pathways (optic nerves [D], Chiasma [E], optic tracts [F]) on axial 3D-DIR reconstruction. OCT showed comparable pRNFL and GCIPL with pRNFL-IETD and GCIPL-IETD (**G**) in normal ranges, confirming the absence of optic nerve lesion. (**H–J**) A patient with RIS presented with 3D-FLAIR hypersignals (**H**) suggesting demyelinating with a PRL on phase imaging (**I**) and CVS on SWI reconstruction (**J**). The yellow arrow is pointing a T2 lesion with PRL and CVS. The blue arrow is pointing another T2 lesion with CVS.

Optic nerves were studied on 3D-DIR and 3D-FLAIR images (using post-acquisition processing: multi-planar reconstruction; Fig. 1). For each optic nerve, the absence/presence, the number and the total length of DIR hypersignal were recorded. If a patient presented several hyperintensities on optic nerve, the different lengths were summed up. MRI images were anonymized and interpreted independently by two trained examiners, J.C.L. and O.O., with 6 and 15 years of expertise in neuroimaging of demyelinating disorders, respectively; intra- and inter-observer agreement for detection of optic nerve lesion on 3D-DIR had been evaluated previously and was excellent.^21-23^ Discordant results were reconsidered to reach a consensus. Whole visual pathways were analysed. The following MRI parameters were collected: the affected optic nerve segments (orbital, canalicular, cisternal and chiasma), involvement of optic tracts and the volumes of T2 lesions within the optic radiations and primary visual cortex (V1) volume.

Brain T2 lesions were segmented on 3D-FLAIR with a semi-automatic method [ITKSNAP 3.6.0 software (www.itksnap.org)]. T2 lesion volume within bilateral optic radiations (OR) was identified with the Julich histological atlas warped from the Montreal Neurological Institute space to the patient 3D-FLAIR space. Primary visual cortex (V1) was automatically segmented on 3D-T1-FFE sequence with the FreeSurfer 7.4.1® software and manually corrected if necessary. The primary visual cortex volume was measured as the sum of right and left V1. Each volume (V1, brain T2 lesions, T2 lesions within bilateral optic radiations) were normalized in relation to the intracranial volume.

Using the 3D-T2*EPI MRI sequence (Fig. 1), PRL and lesions with a central vein sign (CVS+) meeting the NAIMS proposed criteria were assessed on phase images and 3D-SWI multiplanar reconstruction,^24^ respectively. The ‘6 CVS + lesions’ threshold was considered positive if at least six CVS lesions were present in the whole brain^18^ and the ‘40% of CVS + lesions’ was positive if at least 40% of the total white matter lesions were CVS+.^24^ PRL were defined as a complete/incomplete rim of hypointense signal identified on phase images, as recommended.^19^

### Optical coherence tomography

OCT examination was performed with a Spectral Domain-OCT (Spectralis®, Heidelberg Engineering) on the same day as the MRI. Our OCT protocol included a peripapillary scan for measuring pRNFL [12°, 3.4 mm circular scan around the optic nerve with a minimum of 50 automatic real time (ART)] respecting OSCAR-IB criteria^25^ and APOSTEL 2.0 criteria.^26^ Our analysis included measurement of global and temporal pRNFL thicknesses. We also performed macular scan consisting of 25 vertical scans centred on the fovea (minimum of 25 ART). A macular segmentation was performed with HEYEX software version (multilayer segmentation algorithm, Heidelberg Engineering, version 7.0.1) in an anonymized manner. The mean volume [Early treatment diabetic retinopathy study (ETDRS) 6 mm disc] was calculated for the most representative macular layers represented by the macular ganglion cell layer coupled to macular ganglion cell inner plexiform layer (GCIPL) and the macular inner nuclear layer (INL).

### Asymptomatic optic nerve lesion

In our study, optic nerve lesion was investigated using MRI and OCT, not with VEP.

Presence of asymptomatic optic nerve on MRI lesion was retained if at least one T2 hypersignal was detected within the anterior visual pathways. Following recommendations,^27^ presence of asymptomatic optic nerve lesion on OCT was retained if GCIPL inter-eye thickness difference (IETD) was greater than 4 µm (≥4 µm) and/or pRNFL IETD greater than 6 µm (≥6 µm; Fig. 1).

Presence of asymptomatic optic nerve lesion was confirmed if optic nerve MRI was abnormal or if OCT showed a significant IETD on pRNFL or GCIPL as defined above.

### Cerebrospinal fluid analysis

Kappa free light chain (KFLC) was measured by turbidimetry with the analyzer Optilite® (The Binding Site®, Birmingham, UK) using the serum-free light chain immunoassay Freelite® (The Binding Site, Birmingham, UK), according to the manufacturer’s instructions. OCBs were determined by isoelectric focusing using IgG-specific antibody staining (Hydrasys platform; Sebia, Lisses, France).

Intrathecal immunoglobulin synthesis was considered positive if the patient presented with ≥ 2 OCB. For KFLC, the determination of intrathecal immunoglobulin synthesis was evaluated by the calculation of the KFLC index using this formula: [cerebrospinal fluid (CSF) KFLC/serum KFLC]/(CSF albumin/serum albumin). According to unpublished data from the Lille multiple sclerosis centre, a KFLC index of ≥11 was considered as positive to gain specificity over a previously proposed cut-off of ≥8.9.^28^

### Statistical analysis

Retinal thicknesses and volumes were compared between RIS eyes with optic nerve T2 hypersignal [RIS-MRI^pos^] and those without optic nerve T2 hypersignal [RIS-MRI^neg^]) using a linear mixed regression model to take into account the correlation between eyes within the same patient, by including patients as random effect. Difference in least-square means (LSmeans) estimates (RIS-MRI^pos^ versus RIS-MRI^neg^) from linear mixed regression model were reported with theirs 95% confidence intervals (CIs) as effect size. We also used a linear mixed regression models with patients as random effect to study the associations of retinal layer thicknesses (as dependent variables) with optic nerve/brain MRI parameters (as independent variables, included as fixed effects) before and after adjustment on age and gender (both included as fixed effects). Regression coefficients (β) retinal layer thicknesses per unit increase in optic nerve/brain MRI parameters were reported with theirs 95% CIs as effect size. Residual normality of linear mixed regression models was checked by examining the Quantile–Quantile plots.

Comparisons in intereye retinal thickness (pRNFL and GCIPL measures) differences (IETD) between patients with and without optic nerve lesion on MRI were done using Mann–Whitney U test. Finally, we assessed the concordance between IETD criteria (pRNFL-IETD ≥6 µm/GCIPL-IETD ≥4 µm)^27^ and MRI diagnostic of optic nerve lesion by calculating the Cohen’s κ coefficients with theirs 95% CIs.

We did not correct for multiple comparisons given the sample size and the exploratory nature of this study. All statistical tests were done at the two-tailed α level of 0.05 using SAS software, release 9.4 (SAS Institute, Cary, NC).

## Results

### Description of the population

We included 32 patients with a RIS (63 eyes). One eye of a RIS patient was excluded due to ocular melanoma. The median age at baseline MRI was 40 years (IQ range, 37–57). Our population was predominantly female (68.8%). The main reason for MRI was headache (Table 1). Patients with clinical symptoms such as dizziness were clinically evaluated by a neurologist to rule out a possible first clinical event.

**Table 1:** Baseline characteristics of the patients with a radiologically isolated syndrome Values are total no. (%) or median (25th to 75th percentiles)

Baseline characteristics	*n* = 32
Reasons for initial MRI	
Headache, *n* (%)	14 (43.8)
Dizziness, *n* (%)	4 (12.5)
Hypogonadism, *n* (%)	1 (3.1)
Tinnitus, *n* (%)	1 (3.1)
Faintness, *n* (%)	1 (3.1)
Trauma, *n* (%)	1 (3.1)
Parkinsonism, *n* (%)	1 (3.1)
Others, *n* (%)	9 (28.2)
OCT characteristics	
Optic nerve lesion defined by OCT criteria *n* (%)	9/32 (28.1)
MRI characteristics	
Optic nerve lesions *n* (%)	7/32 (21.9)
Spinal cord lesions *n* (%)	18/31 (58.1)
Juxtacortical lesions *n* (%)	25/32 (78.1)
Periventricular lesions *n* (%)	32/32 (100.0)
Posterior fossa lesions *n* (%)	16/32 (50.0)
6 CVS + lesions *n* (%)	24/32 (75.0)
40% of CVS + lesions *n* (%)	22/32 (68.8)
Paramagnetic rim lesions *n* (%)	10/32 (31.3)
Primary visual area volume (mm^3^)	8526 (6981 to 9136)
Optic radiations lesions volume (mm^3^)	280 (78 to 967)
Brain T2 lesions volume (mm^3^)	3108 (1267 to 7626)
CSF	
Intrathecal immunoglobulin synthesis *n* (%)	18/24 (75.0)
Cell count (mm^3^)	0.8 (0 to 1.6)

At baseline, seven patients (21.9%) had optic nerve T2 hypersignal suggestive of demyelinating lesion. Among them, two patients had bilateral optic nerve T2 hypersignal. Eight patients (25.1%) presented optic nerve lesion defined by a GCIPL IETD and/or pRNFL IETD.

Of the 63 eyes included in our analysis, nine optic nerves (14.3%) showed a T2 hypersignal. Among these nine damaged optic nerves, two showed two distinct lesions on the same optic nerve. Median lesion length was 7.0 mm (IQ range, 6.5–8.1). Lesions of the optic nerve were all located in the orbital or the canalicular portion of the nerve (Table 2). None lesion affected the cisternal part, the chiasma or optic tracts.

**Table 2:** Baseline characteristics of patients’ eyes with a radiologically isolated syndrome

	*n* = 63
**Optic nerve lesion on MRI *n* (%)**	9/63 (14.3)
Total length of the optic nerve lesion (mm)	7.0 (6.5 to 8.1) [*n* = 9]
Lesion topography	
Orbital *n* (%)	7/9 (77.8)
Canalicular *n* (%)	4/9 (44.4)
Cisternal *n* (%)	0/9 (0.0)
Chiasmal *n* (%)	0/9 (0.0)
Optic tracts *n* (%)	0/9 (0.0)
Retinal thickness/Volumes on OCT	
Global pRNFL (µm)	96 (90 to 101)
Temporal pRNFL (µm)	65 (60 to 71)
Total macular volume (mm^3^)	8.6 (8.3 to 8.8)
GCIPL volume (mm^3^)	1.91 (1.78 to 2.01)
GCIPL thickness (µm)	67.6 (63.0 to 71.1)
INL volume (mm^3^)	0.93 (0.89 to 0.97)
INL thickness (µm)	32.9 (31.48 to 34.31)

Values are total no. (%) or median (25th to 75th percentiles).

At baseline, PRL were found in 10 patients (31.3%). Twenty-four patients (75%) presented with ≥6 CVS + lesions and 22 (68.8%) presented with ≥40% CVS + lesions. CSF analysis were available for 24/32 patients and 18 (75%) presented with OCB or KFLC index ≥11.

### Asymptomatic optic nerve lesion on MRI versus no optic nerve lesion on MRI

The comparison between RIS-MRI^Pos^ eyes (*n* = 9) and RIS-MRI^Neg^ eyes (*n* = 54) demonstrated significant differences in OCT parameters (Table 3). RIS-MRI^Pos^ eyes presented with lower pRNFL compared to RIS-MRI^Neg^ (median = 87.4 µm versus 96.8 µm, *P*-value = 0.0034). RIS-MRI^Pos^ also presented with lower temporal pRNFL and mGCIPL (respectively, 64.0 µm versus 53.7 µm, *P*-value = 0.0009 and 68.5 µm versus 62.4 µm, *P*-value = 0.0005). There was no difference on INL volume between the two populations. No association was found between quantitative MRI parameters [as optic nerve lesion length, normalized T2 lesions volume within optic radiations and normalized primary visual cortex (V1) volume] and pRNFL, temporal pRNFL and GCIPL (Table 4). No difference was found when we adjusted the analysis for sex and age. INL was the only OCT parameter showing an inverse association with the optic radiation T2 lesions volume. This association remains significant in the age-/sex-adjusted analysis (*P* = 0.041).

**Table 3:** Comparison of the thickness/volume of different retinal layers according to the presence of an asymptomatic optic nerve lesion (*n* = 9) or not (*n* = 54) in a mixed model analysis

	Eyes without optic nerve lesion	Eyes with optic nerve lesion(s)		
OCT parameter	RIS-MRI^Neg^ (*n* = 54)	RIS-MRI^Pos^ (*n* = 9)	Effect Size (95%CI)	*P*
Global pRNFL (µm)	96.8 (94.5 to 99.1)	87.4 (81.8 to 93.1)	−9.4 (−15.5 to −3.2)	**0**.**003**
Temporal pRNFL (µm)	64.0 (60.4 to 67.7)	53.7 (47.0 to 60.4)	−10.3 (−16.2 to −4.4)	**<0**.**001**
GCIPL (µm)	68.5 (67.3 to 69.8)	62.4 (59.3 to 65.5)	−6.1 (−9.4 to −2.8)	**<0**.**001**
INL volume (mm3)	0.940 (0.921 to 0.960)	0.944 (0.908 to 0.981)	0.004 (−0.030 to 0.037)	0.8222

Significant *P*-value are indicated in bold.

Values are least-square means with their 95% confidence interval and the effect size is the corresponding between group-difference estimated from linear mixed regression models including patients as random effect.

**Table 4:** Associations between retinal thicknesses and optic nerve/brain MRI parameters

		Unadjusted		Age-sex-adjusted	
Retinal thicknesses	MRI parameters	β (95%CI)	*P*	β (95%CI)	*P*
Global pRNFL (µm)	Lesion length	−3.02 (−9.42 to 3.39)	0.29	−0.47 (−8.33 to 7.40)	0.88
	Normalized OR T2 lesions volume	−12.1 (−57.1 to 33.0)	0.59	7.7 (−41.5 to 57.0)	0.75
	Normalized primary visual cortex volume	17.1 (−3.6 to 37.9)	0.10	13.33 (−7.6 to 34.3)	0.21
Temporal pRNFL (µm)	Lesion length	−0.61 (−6.69 to 5.48)	0.81	3.57 (−1.57 to 8.72)	0.13
	Normalized OR T2 lesions volume	−3.7 (−47.3 to 40.0)	0.87	16.8 (−31.3 to 65.1)	0.49
	Normalized primary visual cortex volume	10.4 (−9.8 to 30.7)	0.31	7.5 (−13.4 to 28.5)	0.48
GCIPL (µm)	Lesion length	−1.01 (−4.12 to 2.12)	0.46	0.64 (−3.01 to 4.3)	0.65
	Normalized OR T2 lesions volume	−28.0 (−51.9 to −3.8)	0.023	−16.3 (−42.8 to 10.2)	0.22
	Normalized primary visual cortex volume	6.4 (−5.2 to 18.0)	0.28	4.9 (−6.7 to 16.7)	0.40
INL (µm)	Lesion length	0.003 (−0.030 to 0.035)	0.84	0.005 (−0.049 to 0.060)	0.80
	Normalized OR T2 lesions volume	−0.31 (−0.52 to −0.097)	**0**.**005**	−0.25 (−0.48 to −0.010)	**0**.**041**
	Normalized primary visual cortex volume	−0.09 (−0.19 to 0.019)	0.11	−0.07 (−0.17 to 0.034)	0.19

β indicates the regression change (mean change) of retinal thicknesses parameters per one-unit increase in MRI parameters, estimated from linear mixed regression models including patients as random effect.

### Intereye retinal thickness difference to detect optic nerve lesions

In patients presenting optic nerve lesion(s) on MRI (*n* = 7), the median GCIPL-IETD (2.829 µm [IQ range, 0.353–4.951]) and pRNFL-IETD (7.0 µm [IQ range, 1.0–8.0 µm]) were higher than in patients without optic nerve lesion on MRI (*n* = 24; 0.707 µm [IQ range, 0.353–1.415] and 1.5 µm [IQ range, 1.0–4.0 µm], respectively), but statistical significance were not reached (*P* = 0.084 and *P* = 0.11).

If we applied the pRNFL-IETD threshold ≥6 µm, we confirmed optic nerve lesion in four RIS-MRI^Pos^ patients and found optic nerve lesion in two additional RIS-MRI^Neg^ patients. Agreement between pRNFL-IETD and MRI was moderate (κ = 0.52; 95%CI, 0.15–0.89).

If we applied the GCIPL-IETD threshold ≥4 µm, we confirmed optic nerve lesion in two RIS-MRI^Pos^ patients and found optic nerve lesion in two additional RIS-MRI^Neg^. Agreement between GCIPL-IETD and MRI was weak (κ = 0.24; 95%CI, 0.00–0.65).

Combining GCIPL- and pRNFL-IETD, we confirmed optic nerve lesions in five RIS-MRI^Pos^ patients and found optic nerve lesion in three additional RIS-MRI^Neg^ patients. Then, agreement between OCT and MRI remained moderate (κ = 0.57; 95CI, 0.23–0.91).

**Figure 2:**
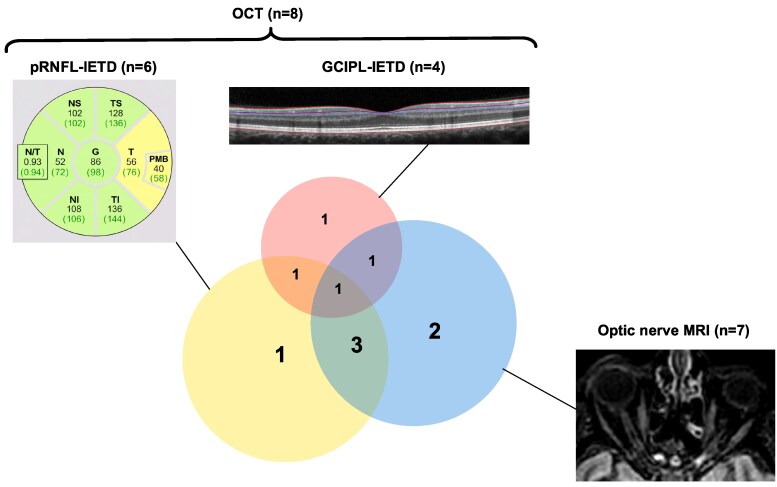
**Schematic representation of patients with RIS with optic nerve lesion(s) according to OCT and optic nerve MRI.** Ten patients presented with optic nerve lesion according to MRI and/or pRNFL IETD ≥6 µm and/or GCIPL IETD ≥4 µm. Only one patient presented an optic nerve lesion according to the three modalities. Six patients presented with optic nerve lesion according to at least two modalities. Four patients presented an optic nerve lesions according to only one modality. We assessed agreement between the techniques by calculating the Cohen’s kappa coefficients with theirs 95% CIs. Agreement between pRNFL-IETD and MRI was moderate (κ = 0.52; 95%CI, 0.15–0.89). Agreement between GCIPL-IETD and MRI was weak (κ = 0.24; 95%CI, 0.00–0.65). Combining both pRNFL-IETD and GCIPL-IETD, agreement between OCT and MRI remained moderate (κ = 0.57; 95CI, 0.23–0.91).

If we considered optic nerve MRI and/or OCT, we demonstrated optic nerve involvement in 10 RIS patients (31.25%; Fig. 2).

### Prognostic value of MS biomarkers

Median clinical follow-up from the baseline to the last clinical evaluation was 22.1 months (IQ range 10.2–34.8). During follow-up, three patients presented with a first clinical event correlated to new gadolinium enhanced lesions. One patient presented during follow-up with a left definite optic neuritis.^29^ At this patient’s baseline MRI, no optic nerve T2 hypersignal was detected. Another patient presented a clinical episode of myelitis. The third patient presented with a subacute cerebellar syndrome with new infratentorial lesion. Two other patients presented with insidious modification of clinical examination and reduced motor performance without relapse suggesting a progressive form of MS. These two last patients had at least two spinal cord lesions at the baseline MRI. One of these two patients with clinical insidious progression had new brain lesions. In total, five patients (15%) converted in clinical MS disease at last follow-up. The median time between baseline and the clinical disease onset was 9.2 months (IQ range, 6.9–17.2). Due to the small number of patients in our RIS cohort, we cannot perform statistical comparative analysis (Table 5) between group with and group without the following baseline characteristics: optic nerve lesion, ≥6 CVS + lesion, presence of OCB in CSF, ≥40%CVS + lesion, ≥1 PRL.

**Table 5:** Distribution of specific biomarkers of multiple sclerosis according to the occurrence of a first clinical event and radiological dissemination in time

	Clinical dissemination in time	
	No (*n* = 27)	Yes (*n* = 5)
Optic nerve lesion (OCT and/or MRI)	7/27 (25.9)	3/5 (60.0)
≥6 CVS + lesions	19/27 (70.4)	5/5 (100.0)
Intrathecal Ig synthesis	15/20 (75.0)	3/4 (75.0)
≥40% CVS + lesions	18/27 (66.7)	4/5 (80.0)
≥1 paramagnetic rim lesions	7/27 (25.9)	3/5 (60.0)

	Radiological dissemination in time	
	No (*n* = 19)	Yes (*n* = 12)
Optic nerve lesion (OCT and/or MRI)	6/19 (31.6)	4/12 (33.3)
≥6 CVS + lesions	12/19 (63.2)	11/12 (91.7)
Intrathecal Ig synthesis	9/14 (64.3)	9/10 (90.0)
≥40% CVS + lesions	10/19 (52.6)	11/12 (91.7)
≥1 paramagnetic rim lesions	7/19 (36.8)	3/12 (25.0)

Proportion of patients having one of these parameters was always higher or equal in the clinically converting group, notably the presence of optic nerve lesion and the presence of at least one PRL. In the clinically converting group, three patients (60%) had an optic nerve lesion defined by OCT/MRI, while in the remaining RIS group (*n* = 27), seven patients had optic nerve lesion (25.9%). In the clinically converting group, three patients (60%) had at least one PRL, while in the remaining RIS group (*n* = 27), seven patients (25.9%) had at least one PRL.

All but one patient had at least one MRI during FU. Among them, 12 presented with new lesion(s) including four of the five patients with clinical conversion. Among these 12 patients, seven had gadolinium enhancing lesion(s). Thus, twelve patients were classified in the MRI converting group. For all tested parameters except PRL, the proportion of patients showing one of the parameters was always greater in the MRI converting group.

### Application of MS criteria

At baseline, 30 patients (93.8%) presented spatial dissemination according to the revised 2017 McDonald MS criteria, three (9.4%) had gadolinium enhanced lesions and 18 (56.3%) presented oligoclonal bands on CSF analysis. Combining these results, 19 (59.4%) RIS patients fulfilled DIS and DIT according to revised 2017 McDonald MS criteria.

At baseline, 26 patients (81.3%) would have fulfilled the 2024 revised McDonald criteria for asymptomatic MS but following recommendations (age > 50 years or vascular risk factors/disease or headache), 21 patients (65.6%) fulfilled the asymptomatic MS diagnostic criteria. At the end of follow-up, by applying these more stringent criteria, 24 patients (75%) fulfilled DIS and DIT according to the revised McDonald criteria. With the use of PRL as an additional feature for MS diagnosis at RIS stage, one additional patient would meet the criteria of asymptomatic MS. This patient had an optic nerve lesion according to OCT, periventricular lesions and CVS6+/CVS40%. She had no spinal cord or infratentorial lesion.

## Discussion

In this study, we found that (i) asymptomatic optic nerve lesions observed on MRI at the RIS stage were associated with lower retinal thicknesses. (ii) Combining OCT and MRI, we reported the presence of asymptomatic optic nerve involvement in a substantial proportion of RIS patients. (iii) OCT and MRI seem complementary for the detection of optic nerve lesion. (iv) More than half of our cohort of RIS patients met the revised MS diagnosis criteria. If PRL was included in asymptomatic MS criteria, one additional patient would have met MS criteria at baseline. (v) The majority of patients converting to clinically definite MS had optic nerve lesion.

In literature, frequency of asymptomatic optic nerve lesion on MRI has not been described. Using the same methodology, we successively investigated the presence, role and consequences of asymptomatic optic nerve lesions in RRMS,^12^ CIS^13^ and RIS in the present study. As expected, the proportion of asymptomatic optic nerve lesions detected by MRI seems lower in eyes of our RIS cohort (14.3%) than in patient’s eyes with a more advanced stage of the disease, such as CIS (18.1%)^13^ or RRMS (32.8%).^12^ At the patient level, we also observed a lower proportion of RIS patients (21.9%) presenting with asymptomatic optic nerve lesion(s) on MRI than at CIS stage (30.8%)^13^ or RRMS stage (42.9%).^12^ The same ranking is observed if we focused on asymptomatic optic nerve lesion length. Using the same MRI sequence on the same 3T MRI scanner, we reported a shorter median optic nerve lesion length (7 mm) at RIS stage than at CIS and RRMS stages, where the mean length of asymptomatic optic nerve lesion is 10 mm and 13.3 mm, respectively, and the mean length of symptomatic optic nerve lesion is 13.8 mm and 20.6 mm, respectively. This increasing lesion length across diseases stages (RIS < CIS < RRMS) could be due to lesions of different ages and a greater degenerative process.^30^ Symptomatic optic nerve lesions are inherently longer and may be more prompt to a degenerative process. In our RIS cohort, lesions detected within the anterior optic pathways were always located at the optic nerve, and mainly at orbital part or canalicular parts. Our findings concur those of previous studies reporting an anteroposterior gradient in MS, irrespective of disease stage.^11,22,30,31^

In MS, optic nerve lesions are associated with neuroaxonal loss.^32^ Global/temporal pRNFL and macular GCIPL thicknesses measured with OCT are significantly reduced in MS^12^ and CIS patient eyes^13^ with an asymptomatic optic nerve lesion. Optic nerve lesions whether they are symptomatic or asymptomatic represents the major cause of neuro-axonal retinal degeneration. In this study, we found similar results at the RIS stage. Eyes with optic nerve DIR hypersignal have lower inner retinal thicknesses (pRNFL, macular GCIPL) suggesting neuroaxonal loss secondary to optic nerve damage. We previously showed a gradual inverse correlation between lesion length and inner retinal thicknesses.^11,12,22^ No association was found between inner retinal thicknesses and the optic nerve lesion length. This may be because of the relatively small size of our cohort, the smaller lesion lengths measured at RIS stage and a more restricted range of values.

No studies at RIS stage have investigated the association between retinal thicknesses and imaging with T2 lesion volume within the optic radiations or primary visual cortex volume. In RIS population, a study with a smaller population of 15 patients showed an association between pRNFL and total brain volume or white matter volume.^6^ Another study interested in white matter lesions number/volume in the whole brain.^3^ These studies did not search for asymptomatic optic nerve lesion. However, as it has been shown that degeneration within the visual pathways is both anterograde and retrograde,^33^ it is important to consider the whole brain and optic radiations to better understand the neurodegenerative process. In our study, the absence of association between inner retinal thicknesses and optic nerve lesion length could be due to retrograde transsynaptic degeneration associated with T2 lesion burden within the optic radiations, but we failed to find any association too. The only OCT parameter that showed an inverse association with the normalized optic radiations T2 lesion volume was the INL. In literature, a thicker INL has been associated with a larger volume of optic radiations lesions in MS patients,^12^ an increased risk of brain T1-gadolinium enhanced and T2 lesions at FU in RRMS^34^ and an increased volume of brain T2 lesions in RIS on longitudinal FU.^7^ In our study, this association is inverted. This link appears to be weak, and aside from a statistical association, we have no clear physiopathological explanation for these findings.

When comparing OCT parameters from our RIS cohort to others, the pRNFL and GCIPL were thicker in two studies.^5,6^ The different versions of segmentation software used may have an influence, but the mean age of 35 and 29 years, respectively, compared to 40 years in our study, could mainly explain this difference. As it is well known, progressive thinning of the pRNFL and GCIPL occurs over time in the healthy population,^35^ in RIS^6^ and in MS patients.^36^ These two studies demonstrated an association between lower GCIPL or pRNFL and the risk of clinical conversion.^5,6^ Because of the sample size, we were unable to assess this association, but in our cohort, a majority of converters had an asymptomatic optic nerve lesion. Furthermore, lower pRNFL and GCIPL thicknesses were found in patients with an optic nerve lesion on MRI. No previous study has searched for optic nerve lesions on MRI in RIS. These asymptomatic optic nerve lesions may represent a confounding bias when attempting to prove the prognostic value of pRNFL and GCIPL thicknesses.

We did not report any substantial agreement between IETD and optic nerve MRI. Our sample size does not enable us to look for the most accurate IETD threshold to detect an optic nerve lesion on MRI. Considering the weak agreement between GCIPL-IETD and optic nerve MRI, we can suppose that the recommended GCIPL-IETD threshold of 4 µm^27^ may not be sensitive enough to detect optic nerve involvement at RIS stage. We had previously proposed a lower optimal GCIPL-IETD threshold (≥1.42 µm) to detect asymptomatic optic nerve lesion in CIS patients with good diagnostic performance (sensitivity 89.3%, specificity 72.6%).^13^ Further studies are needed to define the more adequate retinal layer and the best IETD threshold. Optic nerve lesion length was lower in our RIS cohort and it cannot be ruled out that the optimal IETD threshold is lower in RIS than in CIS. OCT and MRI seem complementary for the detection of optic nerve lesion.

Paramagnetic rim lesions have already been reported in RIS population, with a higher proportion (61–63%)^37,38^ but also with a lower proportion (26.7%).^39^ Using a similar 3D-SWI EPI sequence at 3T, we reported that a third of patients had PRL. We did not measure the exact proportion of white matter lesions showing a paramagnetic rim. The addition of PRL to the diagnosis criteria for asymptomatic MS would enable an additional patient to be diagnosed with MS diagnosis and to start a disease-modifying therapy at a very early stage.

In our study, the proportion of patients with ≥6 CVS or ≥40% CVS + was similar and around two thirds. A study of 20 RIS patients^40^ evaluated the proportion of patients with ≥6 CVS or ≥40% and demonstrated higher proportion (95% and 90%, respectively) than in our study. However, the study population was different, including older patients with RIS diagnosis according to the 2009 RIS criteria which are known to be more stringent.^41^ A recent study evaluating the diagnostic performance of the central vein sign showed similar performance between these two thresholds in RIS.^42^ In line with literature, our results suggest an overrepresentation of patients with >6CVS or ≥40% CVS + in the converting groups, confirming the diagnostic value of this imaging biomarker.

Our study has several limitations. Due to the small number of patients and clinical events, we could not perform statistical comparison between those who converted to MS and those who did not. RIS remains a rare condition, but this may be due to some difficulty in diagnosing RIS, as well as reluctance to perform diagnostic work-up on asymptomatic subjects. Nevertheless, our sample size remains comparable to other studies.^5,6^ Another limit of the study is the heterogeneous duration FU of our cohort due to its retrospective design. Finally, we did not evaluate the visual function but we performed a homogeneous and complete imaging protocol for the visual pathways.

The principal strength of our study lies in the evaluation of optic nerve lesion with a validated and accurate MRI sequence^21,22,43^ correlated with OCT parameters. In addition, we performed a complete structural analysis of the visual pathways and described susceptibility imaging data associated to clinical and MRI FU.

## Conclusions

Our study demonstrated that optic nerve lesions occur in a significant number of patients with RIS. These lesions are associated with lower retinal thicknesses suggesting that it is the main cause of retinal thinning in RIS. These findings confirm that neurodegenerative process occurs early and is measurable at the earliest stage of MS. Optic nerve and paramagnetic rim lesions appear to be more prevalent in patients who clinically convert to MS. However, larger cohorts are needed to confirm their predictive value. In RIS, we need longitudinal multicentre imaging studies to better describe, define and evaluate biomarkers of interest such as retinal thickness measured by OCT and MRI parameters including CVS and PRL. It should help clinicians diagnose MS at a very early stage and better stratify the risk of disease progression in order to treat quickly and efficiently.

## Data Availability

The data that support the findings of this study are available from the corresponding author upon reasonable request.
